# A Few More Words on Pyorrhœa

**Published:** 1895-05

**Authors:** A. V. Elliott

**Affiliations:** Florence, Italy.


ARTICLE V.

### A FEW MORE WORDS ON PYORRHŒA.

BY DR. A. V. ELLIOTT, FLORENCE, ITALY.

In presenting this subject of Pyorrhœa Alveolaris, I wish to say at the outset that I am making no claim to original research, I have not gone into the etiology of the subject, nor into the close scientific hair-splitting questions which are all current to-day in regard to this puzzling and perverse disease. I have left this to men better qualified and equipped for such work, whose tastes, talents and enthusiasm give them a loving and caressing fondness for the habits and social life of the little microbe, who, as the poet has said, "Gets there all the same."

But without having gone into the deepest depths of the study of the pathology of the disease, I have had, in common with each of you, a good deal of practical experience with it,—enough to prove to me that there can be no hard and fast rule to apply to it. Usually when a case is presented to our notice the tooth has changed its position a little and is found to be more or less loose and sometimes elongated. The gum about the tooth is congested and unhealthy looking, and on taking a probe to explore it is found that more or less separation of the tooth from its attachment has taken place—usually more on one side than on the other—and that the process to greater or less degree is absent. Neuralgic pains are often associated with this condition of things—especially towards the latter stages—arising, no doubt, from the nerves in the roots having lost, through the progress of the disease, the natural protection of the gum and process—the root itself not being sufficient to protect the nerve from unusual thermal shock.

I deem it unnecessary in the presence of this audience to describe further this disease. What we want to know as practicing dentists is what is it, what is the cause, and how to cure it. Some of the best men in our profession are in open antagonism in regard to its origin, one set holding to the constitutional or systemic, the other to the local and limited. The constitutionalists agree that the disease, as we find it in the mouth, is but the expression of some fault or vice in the blood such as uric acid, scurvy, struma, etc. The uric acid or gout theory is very plausible in spite of the fact that for many years gout has been made the scapegoat of obscure diseases in general, and has been generally used as a convenience to cover up ignorance on the part of the diagnosticians.

In justice to Prof. Pierce, however, in this connection we must admit that his conclusions have been arrived at after a careful and scientific examination of the subject, and deserves our serious attention. It may be, and very probably is, the case that vitiated blood has more or less to do with the disease—whether primarily or secondarily is the question,—whether or not the disease has its origin in the blood. We, as dentists, have to devote our principal energies to the local conditions. They require the most careful attention, both mechanical and chemical, to insure any promise of amelioration.

What makes the disease so perplexing is the apparent perverseness of it—so contradictory. The "Eureka" of yesterday gives place to the discouragement of to-day. Rheumatic and non-rheumatic, gouty and non-gouty, strong and healthy, weak and anemic,—the only exception to this omnibus of conditions is age, we do not often find it in the young. Before Riggs' time there was not that close study into the pathology of the human jaw, we are favored with in these days of dental conventions. Our occupation was then more mechanical than professional and some even now, I am sorry to say, seem to consider it so.

It is easy to call the attention to the fact that the ancient and primitive men had perfect teeth, and so all things considered they should have had, but what proof have we of the fact. As far as my observation has taught me there seems not to be so much difference as the changed conditions of living would warrant. I have studied the jaws of the ancient Egyptians at the museums in Cairo, the lake dwellers here in Switzerland, the ancient Romans at Pompeii, and in the Catecombs; the ancient Britons, the mound dwellers at Harvard University, the Etruscans in Italy; and but recently had the pleasure of seeing, through the courtesy and guidance of my friend Dr. Dunn, of Florence, a skull whose age it is impossible to estimate. It was so well preserved by the process of petrification that on removing a portion of that which was formerly the skull, the petrified dura mater showing the convolutions of the brain and the superficial blood vessels were still to be seen.

Yet this most ancient of earth's inhabitants evidently suffered from tooth-ache, evidenced by two badly decayed teeth and a root. This interesting skull is in the museum of San Gimignano, a walled and medieval city, situated among the mountains of Central Italy. It was found several meters under the surface, in a strata of the teretary period associated with the disintegrated rock, fish shells, rubble, etc., the whole of which, although now over 1,000 meters above the sea level, was once under water and that for unknown ages.

Of the great number of ancient skulls which I have had the opportunity of examining I have invariably found a certain percentage among them bearing evidence of decay of the teeth, thus proving our remote ancestors were not so very different from ourselves as regards immunity from diseases of the masticating organs. If we go still further back past the missing link, which at present divides us from the brute creation, we find that the gorilla and chimpanzee—our nearest relations the other side of that link—are better off than ourselves in this respect. So are these animals as we find them now. The gorilla has a magnificent set of teeth, including powerful cuspids and third molars.

One would suppose that in those earlier times referred to, when a less complicated civilization existed to weaken men, and especially when the survival of the fittest was in vogue—the weaklings having gone to the wall in early life,—that decay of the teeth would be almost impossible and the same in regard to the disease we are now considering. I am sorry not to be able to indicate definitely the ancient skulls which I have seen showing unmistakable evidence of the ravages of this disease.

In examining these ancient skulls referred to, I was then interested in the question of caries and alveolar abscess and neglected other matters. But in thus referring in general terms to decay of the teeth I wish to assume by analogy other lesions relating to the masticating organs as well, so as to emphasize the immutable law in Nature, namely, *a given set of conditions invariably produce a given result.* Everything in nature is but a problem in chemistry or mathematics. If we knew all the figures of the proposition or the chemicals in the compound and had the ability we could solve the problem, or by qualitative and quantitative analysis get at the result. But in this, like other problems in nature, we need more data before we can arrive at the truth. A solution will come in good time. I have faith in the Millers and Huxleys of the future as well as in those of the present day.

In my opinion pyorrhœa alveolaris is but the result of a tendency in the original constitution acted upon and developed by favorable conditions. I have had this truth strongly impressed upon me by observing the relative difference there exists between people of the same race and condition in life, but living differently. Take for example, the Italian peasants. Those of the country whose diet is simple, consisting of polenta and hard black bread with hardly any meat and no condiments, have good and strong teeth and healthy gums, while their relatives, those of the cities, many of them originally from the country, living under different sanitary and dietary conditions have usually disgusting mouths. Theirs is a mixed diet of poor quality, including in it all the extras they can afford. But even among those, all are not equally affected. Some have moderately clean mouths.

I think we will always find that those races which are blessed with good teeth and healthy gums live on a simple and natural diet. Even our Irish when they first land at Castle Garden, before they become the aldermen and mayors of our cities, have generally good white teeth. Their diet at home was simple enough, consisting mostly of potatoes and milk—and good fighters they are, too. The

Arabs have also very good teeth. Their diet is likewise very simple.

I was recently told by a gentleman fresh from among the blacks of South Africa that the Zulu Kafirs there, the natives of the place, have in their native state strong and sound teeth, but that those who are brought into contact with the white man and his civilization are much worse off, and frequently resort to the white chemist and his forceps for relief.

There are two great principles we must recognize as potentialities in regard to the teeth and their environment,—the way people live and the care they bestow on their teeth. A bad way of living induces bad blood and impure secretions. This idea favors the constitutional side of the question, but it is true, nevertheless. Rich people and those most favored, to outward appearance, often do violence to nature both as to quality and quantity in what they eat. Nature will not suffer violence without revenging herself sooner or later.

A favoring condition in those predisposed is the neglect to give the teeth the exercise they require. Some teeth need more exercise than others. It would seem as if in the combination of teeth and jaw strong teeth were often placed in jaws too delicate for their proper support. The teeth are all right, but the jaw is deficient. What is needed in such cases is to brace up the jaw by giving it plenty of exercise—gymnastics and massage. It is said our forefathers had larger jaws than we have. They probably ate tougher steak and had less delicate French cooking.

Even dogs, if fed on soft and rich food without the opportunity of exercising their teeth, tearing the meat from the bone, etc., will suffer in time with the ills the superior animal, man, has, generally speaking, the monopoly of.

It is a well known fact in medicinal science that the non-use of an organ weakens it and that giving it proper exercise strengthens it. An arm done up in a sling soon shrinks away and an arm at the blacksmith's anvil will develop great power. With myself I sometimes notice a tenderness in certain teeth when commencing to masticate with them after having given them a holiday for some time by chewing on the other side, Whenever I discover the tenderness I take the hint and work that side for all it is worth until the tenderness disappears. You know it is a common habit with us to use one arm more than the other—some people are almost blind in one eye without knowing it—from not using it.

Another factor in encouraging this disease is improper cleaning of the teeth and gums. A perfunctory, careless, and slovenly method is almost as bad as total neglect. The gums about the neck of the teeth should have a thorough massage with the tooth brush. The ideal mouth. judging from an extended experience, is that of the average American young lady. In her mouth, the gums are firm and healthy, the teeth free from tartar and the breath sweet. She owes it all to her innate sense of refinement and her tooth brush. She may be a tooth brush crank but it pays her.

In regard to the treatment of and prognosis there are many cases presenting themselves which are hopeless—too far gone to attempt anything but alteration and with most of those the best remedy is the forceps.

There are others whose general appearance discourages any prolonged effort.

There are patients who never have the desire or patience to give the time demanded. After eliminating those we have a great variety to try our luck upon, from the doubtful to the hopeful, according to the stage of the disease and the patient's powers of co-operation.

The first and most important essential is scaling to remove all foreign adhesions, then a thorough washing out of the pockets and around the roots with peroxide of hydrogen—sometimes adding a little of the 1,000 solution of the bichloride of mercury. After a little rest and rinsing out the mouth, the next application should be of the nature of a stimulant and I know of nothing better than a one in 10 solution of sulphuric acid with a few drops of the tincture of iodine added. This can be injected into the pocket or pockets under the free margin of the gums with a Donaldson bristle having a few shreds of cotton wrapped about it. This treatment might be changed sometimes and Robinson's remedy or something else substituted. The patient on leaving after the first visit should be instructed in the manner of using his brush, and as to the kind of brush. A prescription for a disinfecting mouth wash should be given him. The one I would give is Prof. Millar's saccharin mixture, which should be used diluted. A formula for a simple and medicated tooth powder should also be given, such as prepared chalk, orris root and chlorate of potash in equal parts. Besides which he must be instructed to rub the gums about the roots with the fingers, especially up and down, a sort of massage exercise strengthening the gums and tending to press out foreign matter from the pockets. And finally he should be en couraged to exercise the loose teeth as much as possible by chewing on them freely and persistently. Let him chew gum or anything else except tobacco, in which case the remedy would be worse than the disease. Like one's experience in gymnastics—at first the muscles are tender and easily fatigued, after awhile the exercise is a pleasure.

On my way here yesterday I passed a stone quarry. The size of the mallets used by the workmen were enormous—half an hour's work with one of them would use me up for a day.

In regard to the constitutional treatment my advice is to refer the client to the family physician. Proper treatment of the disease requires much co-operation from the patient.—*Proceedings of American Dental Society of Europe.*

